# Reversible Conversion of Odd/Even One-Way Modes in Magneto-Optical Photonic Crystal Double-Channel Waveguides

**DOI:** 10.3390/nano12142448

**Published:** 2022-07-17

**Authors:** Xinyue Yu, Suna Zhuang, Jianfeng Chen, Zhi-Yuan Li, Wenyao Liang

**Affiliations:** 1 School of Physics and Optoelectronics, South China University of Technology, Guangzhou 510640, China; alyssayuu@163.com (X.Y.); zhuangsuna0712@163.com (S.Z.); phjfchen@mail.scut.edu.cn (J.C.); phzyli@scut.edu.cn (Z.-Y.L.); 2 State Key Laboratory of Luminescent Materials and Devices, South China University of Technology, Guangzhou 510640, China

**Keywords:** magneto-optical photonic crystals, one-way modes, mode conversion, waveguides

## Abstract

We have studied the transmission properties of odd/even one-way modes and their reversible conversion in a double-channel waveguide consisting of two magneto-optical photonic crystals (MOPCs) sandwiched with Al_2_O_3_ PC. There exist two pairs of even and odd modes, i.e., M1(even)/M2(odd) or M3(odd)/M4(even) modes, for the double-channel waveguides with one- or two-stranded coupling layer of Al_2_O_3_ rods, respectively. Among them, the M1, M2, and M3 modes are caused by the weak coupling strength of two sub-waveguides, while the M4 mode results from the strong coupling effect and supports dispersionless slow-light propagation. Furthermore, we realize the reversible conversion between odd and even modes (i.e., between M1 and M2 modes, or M3 and M4 modes) in the one- or two-stranded structure, respectively, by adjusting the length and position of the perfect electric conductor (PEC) defect properly to cause the desired significant phase delay along the upper and lower equivalent transmission paths. Additionally, we find that the robustness of the M1 even mode is poor because of extra excitations of counter-propagation modes near the right Brillouin boundary, while the other three modes have extremely strong robustness against PEC defects and their one-way transmittances are nearly 100%. These results hold promise for many fields, such as slow-light modulation and the design of topological devices.

## 1. Introduction

In recent years, there has been an increasing interest in topological photonics which supports unidirectional electromagnetic wave propagation and show robustness against backscattering, disorders, and defects [1,2,3,4,5]. Researchers have found that one-way edge modes can appear in valley PCs and magneto-optical photonic crystals (MOPCs) [2,3,4]. Among them, MOPCs provide an important platform for studying the generation and transport properties of topological photonic states (TPSs) by breaking time-reversal symmetry via a *dc* external magnetic field (EMF), which can be seen as a one-way mode [6,7,8]. In 2008, Raghu and Haldane first predicted the existence of electromagnetic one-way edge states as analogs of the quantum Hall effect in two-dimensional (2D) PCs possessing Dirac point-derived bandgaps [9,10]. Later, Wang et al. experimentally verified the one-way edge modes using 2D MOPCs in the microwave regime and observed robust one-way transmission against backscattering [11]. MOPCs have been used to realize various transmission behaviors, including slow-light [12,13,14], topological light trapping [15], and antichiral edge states [16,17].

Extensive research has shown that the TPSs based on MOPCs can be used to overcome the back-scattering problem because of topological protection in edge states. Therefore, a considerable amount of work on topological devices to realize the manipulation of light propagation, such as one-way waveguides, power splitters, and optical switches, were proposed [18,19,20,21,22]. In 2009, Chong et al. experimentally realized one-way modes in square lattice MOPCs [11]. In 2010, Chen et al. proposed a scheme to realize a tunable one-way cross-waveguide splitter based on a one-channel waveguide constructed with MOPC and conventional PC [21]. In 2012, Liu et al. investigated TPSs propagation characteristics in the triangular lattice MOPC plate [19]. In 2016, Gao et al. realized topological photonic edge states in a designer surface plasmon platform [23]. In 2017, Chen et al. exhibited multiple topological phases with the design of a tunable two-dimensional MOPC [24]. In 2019, Chen et al. designed a switchable slow light rainbow trapping state generated by a strongly coupled TPS in a photonic system made from an MOPC waveguide channel [12]. In 2020, Yang et al. numerically investigate the details of the disorder-induced topological state transition in a Chern insulator composed of PC [25]. In 2022, Chen et al. predicted and observed robust one-way bulk states in MOPC using the heterogeneous Haldane model [26].

Most previous research to generate TPSs, especially that using chiral edge states in a 2D photonic crystal system, is based on single-channel waveguide constructed by two structures with different topological invariants and configurations sharing a common band gap. Typical single-channel waveguides include MOPC-air-MOPC, topologically nontrivial PC-air-trivial PC, and topologically nontrivial PC covered with a metal layer in which the EM wave can transport with high transmittance unidirectionally. In 2021, our group proposed a double-channel MOPC waveguide to achieve a zero GVD slow-light state with topological protection by the strong coupling effect of two one-way modes [27]. So far, to the best of our knowledge, there has been little research about double-channel one-way waveguides where EM waves can propagate unidirectionally along the waveguide and may cause interesting phenomena because of the interaction of sub-waveguides. It is highly desirable to construct double-channel MOPC waveguides with more structural parameters, inheriting properties of conventional TPSs, and a possible coupling effect between two sub-waveguides, which provides a versatile scheme to implement more functionalities, such as the reversible conversion between odd and even modes to be studied here.

In this work, we propose a double-channel MOPC waveguide to study the physical mechanism of the topological waveguide modes and the reversible conversion between odd and even modes resulting from their interaction between two sub-waveguides. There exist M1 (even) and M2 (odd), or M3 (odd) and M4 (even) modes for the one- or two-stranded MOPC waveguides, respectively. Such four modes originate from different coupling strengths of TPSs in two sub-waveguides of the structure. Especially under appropriate parameter conditions, reversible conversions between M1 (even) and M2 (odd) modes, or M3 (odd) and M4 (even) modes are realized for the one- or two-stranded structures, respectively. The robustness of these modes against perfect electric conductor (PEC) defects is also analyzed.

## 2. Materials and Methods

**Figure 1:**
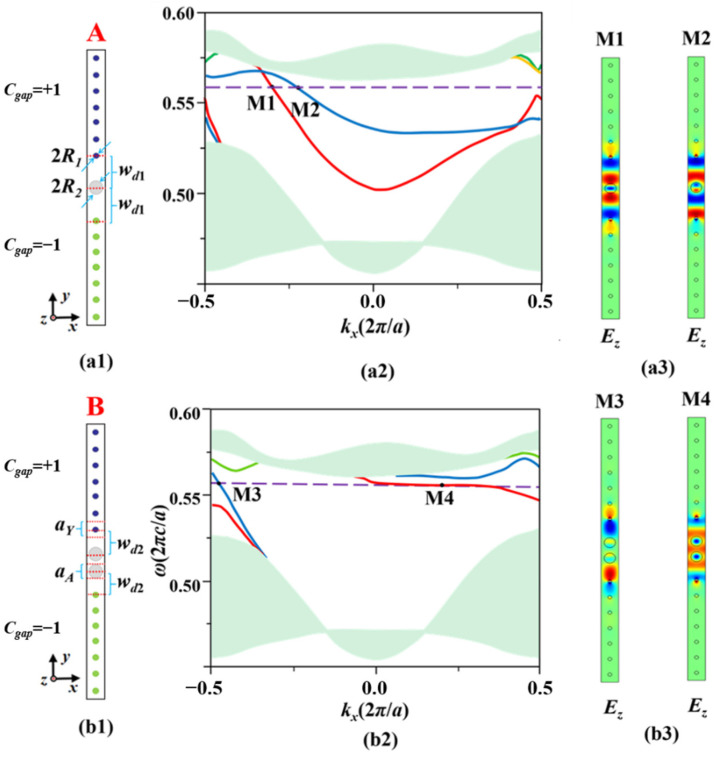
(Color online). (**a1**,**b1**) The supercells of the double-channel MOPCs A and B created by introducing a one- and two-stranded Al_2_O_3_ PC coupling layer into the MOPC structures; (**a2**,**b2**) The band structures for TM polarization corresponding to structures A and B, respectively. The red, blue, green, and yellow lines present four waveguide modes supported in the double-channel MOPCs; (**a3,b3**). The eigenmodal field distributions (*E_z_*) corresponding to M1, M2, M3, and M4, respectively.

We construct two symmetrical double-channel waveguides named A and B by introducing a one- or two-stranded Al_2_O_3_ PC as the coupling layer into the square lattice MOPC. The corresponding supercells are shown in Figure 1(a1,b1), respectively. Both the MOPC and the Al_2_O_3_ PC have the same lattice constants, i.e., *a*_Y_ = *a*_A_. The upper and lower parts are two MOPCs formed by YIG rods with radii of *R*_1_ = 0.11*a* arranged in a square lattice, and they are applied with the same strength of magnetic field *H*_0_ = 1543*Gs* but with opposite directions along the *z* axis, respectively. The radius of the Al_2_O_3_ rods in the middle coupling PC is *R*_2_ = 0.32*a*. The relative dielectric constants of YIG and Al_2_O_3_ rods are *ε*_1_ = 15 and *ε*_2_ = 8.9, respectively. The widths of the sub-waveguides of A and B are *w_d_*_1_ = 2.02*a* and *w_d_*_2_ = 1.52*a*, respectively. With a *dc* EMF *H*_0_ applied in the +*z* direction, the gyromagnetic anisotropy is strongly induced to break time-reversal symmetry and the relative permeability is a tensor as follows:

(1) $$\hat{\mu }=\left(-\begin{array}{ccc} \mu_{r} & j\mu_{k} & 0\\ j\mu_{k} & \mu_{r} & 0\\ 0 & 0 & 1 \end{array}\right)$$

where *μ_r_* = 1 + *ω_m_*(*ω*_0_ + *jαω*)/[(*ω*_0_ + *jαω*)^2^-*ω*^2^], *μ_k_* = *ωω_m_*/[(*ω*_0_ + *jαω*)^2^-*ω*^2^]. Here, resonance frequency: *ω*_0_ = 2π*γH*_0_, the gyromagnetic ratio: *γ* = 2.8 MHz/Oe, *ω_m_* = 2π*γM*_0_ is the characteristic circular frequency with *M*_0_ being the saturation magnetization, and *α* = 0 is the damping coefficient. We calculate the dipersion relation of waveguide, as well as the Chern numbers of each MOPC. The Chern number of the *n*th band is defined as:

(2) $$C_{n}=\frac{1}{2\pi i}\iint_{Bz}\left(\frac{\partial A_{y}^{n}}{\partial k_{x}}-\frac{\partial A_{x}^{n}}{\partial k_{y}}\right)d^{2}k$$

where ***A****^n^*(*k*) = <***E****_nk_*|∇*_k_*|***E****_nk_*> is the Berry connection with ***E****_nk_* the normalized eigenstate for the *n*th band at *k* point, and <***E****_nk_*|***E****_nk_*> = 1. The calculated *C_gap_* for the upper and the lower MOPCs are −1 and +1, respectively.

Most of our work was designed and simulated with the aid of the commercial finite-element analysis package COMSOL Multiphysics. Simulations, including dispersion relation analysis, are carried out in the frequency domain and only the transverse magnetic (TM) modes are considered throughout this work. Floquet periodic boundary and scattering boundary condition conditions were used to simulate the eigenmode-field distribution and propagation behavior for TM polarization corresponding to the sandwiched structure we designed.

## 3. Results

### 3.1. Dispersion Relation Analysis of Double-Channel Waveguide

Firstly, we investigate the dispersion relations of the double-channel MOPC waveguides A and B. The dispersion relations are calculated by the finite element method (FEM) and shown in Figure 1a,b, respectively. For waveguide A with a one-stranded layer of Al_2_O_3_ rods, there appear four waveguide modes, as depicted by the red, blue, green, and yellow lines. Especially within the band gap, there exists a small frequency range from 0.5535 to 0.5657(2π*c*/*a*) where one-way modes can be excited. Without loss of generality, we choose a typical frequency *ω_s_* = 0.5572(2π*c*/*a*) depicted by the dashed horizontal purple line in the band gap to intersect with the red and blue bands at the M1 and M2 points, respectively. The slopes of the M1 and M2 modes are negative and relatively large, implying that the EM waves corresponding to the M1 and M2 modes propagate with a large group velocity unidirectionally.

**Figure 2:**
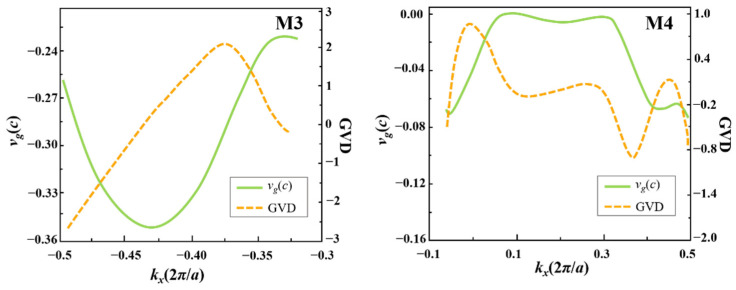
The green and yellow curves represent *v_g_* and GVD for the M3 and M4 modes, respectively.

Likewise, we proceed to study the characteristics of waveguide modes in waveguide B. As shown in Figure 1b, the frequency range of one-way modes in waveguide B is from 0.5354 to 0.5621(2π*c*/*a*) within the band gap, which is much larger than that of waveguide A. The typical frequency *ω_s_* = 0.5572(2π*c*/*a*) intersects with the blue and red curves at points M3 and M4, respectively. The left part of the blue curve is straight-like with a large negative slope, indicating the M3 mode is a one-way mode with a large group velocity. Differently, for the right part of the red curve, one can find that within the frequency range of [0.5354, 0.5621](2π*c*/*a*), the red curve flattens out and continues to decline with increasing *k_x_* and its slope is always negative. Specifically, the slope at point M4 is almost zero, meaning that a one-way slow-light effect can be realized for the flat part of the red curve. We calculate two important parameters, group velocity (*v_g_* = *dω*/*dk_x_*) and group velocity dispersion [GVD = *d*(1/*v_g_*)/d*ω*], numerically, to characterize the property of the M4 mode, as shown in Figure 2.

As seen in Figure 2, obviously, there is a significant difference between *v_g_* for the M3 mode and *v_g_* for the M4 mode, with the M3 mode being a fast one-way mode and the M4 mode being a slow-light state, especially in the *k_x_* range of [0.06, 0.3](2π/*a*). Meanwhile, the M4 mode possesses small volatility around GVD = 0 in the same range of *k_x_*, meaning that the GVD for one-way slow-light transmission in M4 mode is almost zero. It should be noted that the green and yellow band curves in Figure 1(a2,b2) locate outside the bandgap and thus cannot excite by *ω_s._* In other words, the modes located at these two band curves have no influence on the M1, M2, M3, and M4 modes. Therefore, we will only focus on the red and blue bands in the following study.

Next, we study the physics mechanism of these four modes for waveguides A and B from eigenfield characteristics analyses. Figure 1(a3,b3) presents the eigenfield distributions of (M1, M2) and (M3, M4) modes, respectively. For waveguide A, the M1 mode is an even mode which can be seen as a pair of odd sub-waveguide modes symmetrically located in the upper and lower sub-waveguides out of phase, while the M2 mode is an odd mode consisting of a pair of odd sub-waveguide modes distributed antisymmetrically in phase. It is noted that the eigenfields of the M1 and M2 modes mainly concentrate on both sides of the upper and lower sub-waveguide edges (i.e., around the Al_2_O_3_ rods and the edge of YIG rods), and there is only a small portion of field inside the middle one-stranded Al_2_O_3_ PC rods, exhibiting almost no destruction to the original eigenfield distribution in each sub-waveguide. These results imply that both the even M1 and odd M2 modes result from the weak interaction between the sub-waveguides via the middle one-stranded Al_2_O_3_ coupling layer.

Differently, for waveguide B with a two-stranded Al_2_O_3_ PC layer, the left M3 mode is an odd mode that consists of a pair of odd-like sub-waveguide modes distributed antisymmetrically, while the right M4 mode is an even mode which is composed of a pair of odd-like sub-waveguide modes symmetrically. The electric field of the M3 mode is almost completely distributed in the two sub-waveguides (*w_d_*_1_ and *w_d_*_2_) but rare in the Al_2_O_3_ PC coupling layer, meaning that the M3 mode is caused by the very weak interaction of two odd-like sub-waveguide modes, and it supports fast-light transmission in the double-channel structure. On the contrary, the eigenfield of the M4 mode is almost completely concentrated in the Al_2_O_3_ PC coupling layer and the electric fields in the upper and lower parts are symmetrical, revealing that the two sub-waveguide modes have strong interaction in the process of propagating to cause the extreme slow-light state of the M4 even mode, as shown in Figure 2.

### 3.2. Transmission Characteristics of Odd/Even One-Way Modes in Double-Channel Waveguides

**Figure 3:**
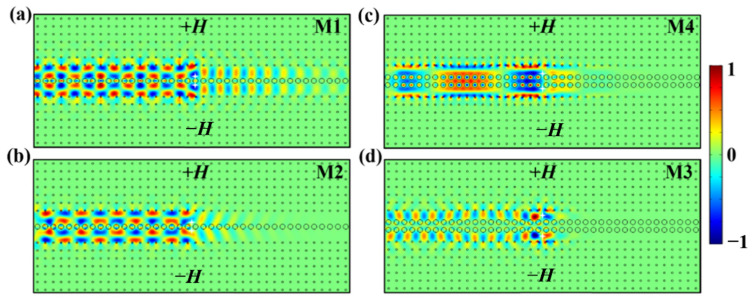
The *E_z_* field distributions for (**a**) M1 even mode, (**b**) M2 odd mode, (**c**) M4 even mode, (**d**) M3 odd mode in double-channel waveguide. The white stars represent the two point sources.

Now, we proceed to study the transmission behaviors of the M1, M2, M3, and M4 modes in the double-channel magneto-optical PC waveguide. We set two point sources oscillating at *ω_s_* with different phase conditions in the center of each sub-waveguide to excite two groups of odd/even modes (i.e., M2/M1, M3/M4) in waveguides A and B. When the initial phases of both point sources are the same, and an even mode is excited; while the two point sources have opposite initial phases, an odd mode is excited instead. Figure 3a,b are the simulation results of the M1 and M2 modes for waveguide A with a one-stranded layer of Al_2_O_3_ rods, while Figure 3c,d are those for the M4 and M3 modes for waveguide B with a two-stranded layer of Al_2_O_3_ rods. For waveguide A (i.e., the one-stranded structure), the energy distributions of the M1 even mode and M2 odd mode in the Al_2_O_3_ PC coupling layer are very weak. Because of such a weak coupling effect, the *E_z_* fields of the M1 and M2 modes are mainly distributed along the edges of two sub-waveguides, leading to the fast-light transmission of EM waves for these two modes. Contrarily, the case for waveguide B is different due to the particularity of the two-stranded structure and the shorter distance between the neighboring Al_2_O_3_ and YIG rods (i.e., the waveguide width becomes smaller). There exists a slow-light even mode (i.e., the M4 mode) and a fast-light odd mode (i.e., the M3 mode), which result from the strong and weak coupling effects, respectively. The simulation results in Figure 3 agree well with the eigenfield analysis in the above discussion in Section II.

Moreover, we notice that the M2, M3, and M4 modes propagate leftwards unidirectionally with a transmittance of almost 100% (Figure 3b–d). However, Figure 3a shows that for the M1 mode, there exists a weak electric field on the right side of the two point sources, indicating that part of the EM wave propagates rightwards. This particular phenomenon can be explained by the band structure in Figure 1(a2), where the right part of the red band curve near the Brillouin boundary is very close to *ω_s_*. In simulations, it is difficult to excite a single frequency exactly. Instead, it will usually excite the EM waves within a certain narrow bandwidth around *ω_s_*. For this reason, when we excite the M1 mode oscillating at *ω_s_*, most of the energy is carried by the M1 mode to propagate leftwards. Meanwhile, it inevitably causes partial excitation of the red band curve near the right Brillouin boundary, leading to a little right-transmitted energy.

### 3.3. Robustness Analysis of One-Way Waveguide Modes

#### 3.3.1. Robustness of M1, M2, M3, M4 Modes

**Figure 4:**
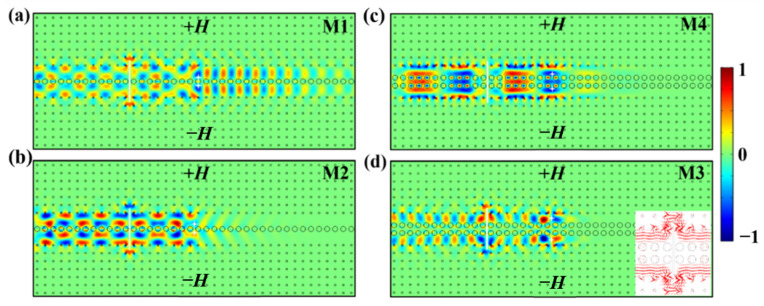
The *E_z_* field distributions when a PEC defect of 5*a* is vertically and symmetrically inserted in the double-channel waveguide for (**a**) M1 mode, (**b**) M2 mode, (**c**) M4 mode, (**d**) M3 mode. The white block inset in (**d**) is the Poynting vector distribution for the M3 mode. The size and direction of the red arrow characterize the strength and propagation direction of EM wave.

So far, we have demonstrated the one-way transmission property of TPSs in the one- and two-stranded waveguides. Next, we studied the transport robustness of these modes against perfect electric conductor (PEC) defects. We inserted a PEC defect with a size of 5a×0.1a vertically and symmetrically into the center of the double-channel waveguide to block EM wave propagation. All other parameters are the same as those in Figure 3, and the simulation results against PECs are shown in Figure 4.

Obviously, the PEC defect hardly has an impact on the power transmittances of the M2, M3, and M4 modes. As shown in Figure 4b–d, the distributions of *E_z_* field almost stay the same before and after bypassing the PEC defect as the EM waves propagate leftwards from the source. We take the case of M3 mode as an example for further discussion. As shown in the enlargement of Figure 4d, the insertion of the PEC defect produces two equivalent routes for the EM wave to go around the two ends of the PEC defect between the YIG rods and the PEC wall. In the upper MOPC, the energy flow rotates counterclockwise to climb over the upper end of the PEC defect and then goes to the left side of the upper sub-waveguide, while in the lower MOPC, the energy flow rotates clockwise to bypass the lower end of the PEC defect and then flows into the lower sub-waveguide. After that, the energy flows in the upper and lower sub-waveguides couple with each other to continuously propagate leftwards, maintaining almost unidirectional propagation. The cases of the M2 and M4 modes are similar to that of M3. These results indicate that the M2, M3, and M4 modes are topologically protected, showing that TPSs possess strong robustness against PEC defects, which is widely used in practice for unidirectional waveguide fabrication. It should be noted that for the M1 mode, because certain bidirectional modes of the red band curve near the right Brillouin boundary are excited simultaneously, the propagation of the EM wave is affected by the PEC defect evidently, exhibiting weak robustness against PEC defects for the M1 mode. Besides, we notice that the *E_z_* field distributions on both sides of the PEC are almost in phase, implying that the path length of 2.5a×2 = 5*a* will cause a phase delay of about 2*k*π (*k* is an integral).

#### 3.3.2. Phase Effect of PEC on Waveguide Mode

**Figure 5:**
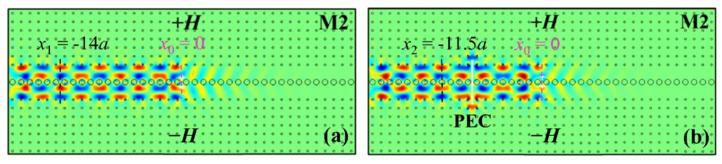
The simulation results for the M2 mode: (**a**) without a PEC defect; (**b**) with a PEC defect (5a×0.1a) vertically and symmetrically inserted in the waveguide. *x*_0_ = 0 is set at the position of the two point sources as denoted by the dashed purple line, while *x*_1_ and *x*_2_ denote the chosen minimum amplitude positions of the two cases.

Next, we discuss the influence of phase effects caused by PEC on TPSs. As seen above, the EM waves propagate leftwards and bypass the PEC defect, which causes a nonnegligible phase delay to sub-waveguides accordingly. Since the *E_z_* distribution is obtained by solving the eigenvalue of the Bloch equation, the phase delay can be roughly confirmed by comparing the modes of TPSs between the normal waveguide (Figure 3) and the waveguide with PEC insertion (Figure 4). We take the M2 mode as an example to demonstrate in detail without loss of generality. Figure 5 compares the simulation results for the M2 mode without or with a PEC defect. In Figure 5a, the point source is placed at *x*_0_ = 0, and *x*_1_ = −14*a* is a reference position with a maximum amplitude of |*E_z_*| field along the black dashed line for the waveguide without PEC defect. According to the difference between the neighboring maximum amplitude positions of the |*E_z_*| field with the identical distribution as *x*_1_ = −14*a*, the Bloch wavelength is approximately *λ_B_* = 4.5*a*. In Figure 5b, due to the phase delay introduced by the insertion of the PEC, this corresponding amplitude position moves to *x*_2_ = −11.5*a*. Therefore, the phase delay caused by the PEC of 5*a* is about 2.5/4.5 × 360° = 200°. It can be concluded that the PEC defect will result in a significant phase delay the longer it exists. With the help of this property, there is a prospect of modulating the phase of one-way modes and designing topological phase-delay devices in the appropriate way as desired. It should be noted that since the PEC is inserted vertically and symmetrically in the waveguide, it causes the same phase delays for the two equivalent paths across the upper and lower ends of the PEC defect, maintaining the same electric field distribution after the EM wave propagates around the PEC defect. Therefore, in these symmetrical cases, the PEC has negligible influence on the odd/even property of the TPS in the waveguide.

### 3.4. Reversible Mode Conversion between Odd and Even Modes in the Double-Channel Waveguides

#### 3.4.1. Odd/Even Mode Conversion

**Figure 6:**
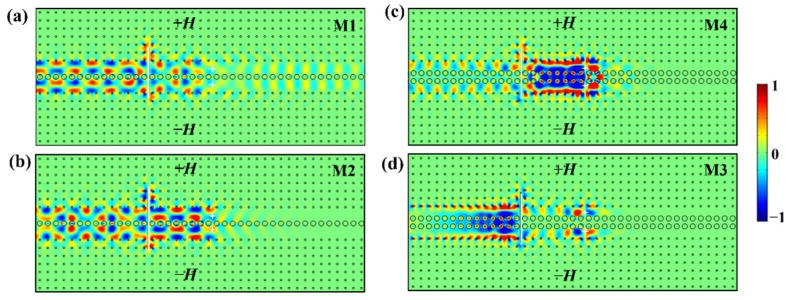
The *E_z_* field distributions with a PEC defect of 6.25*a* vertically and asymmetrically inserted in the double-channel MOPCs waveguide for excited (**a**) M1 mode, (**b**) M2 mode, (**c**) M4 mode, (**d**) M3 mode.

Now we proceed to study the reversible conversion between odd and even modes in double-channel waveguides with asymmetrical insertions of PEC defects. Based on the previous analysis, we extend the upper part of the PEC defect by 1.25*a* (i.e., the length of the PEC defect is increased from 5*a* to 6.25*a*), and the simulation results for M1, M2, M3, and M4 modes are shown in Figure 6.

Obviously, the *E_z_* field distributions of the four waveguide modes on the left and right sides of the asymmetrical PEC have a significant difference as compared with those of the symmetrical cases in Figure 4. Concretely, in waveguide A with a one-stranded coupling structure (Figure 6a,b), we excite the M1 even mode and the M2 odd mode separately. After bypassing the asymmetrical PEC, the EM field distribution of the M1 even (or M2 odd) mode turns into that of the M2 odd (or M1 even) mode, indicating that the M1 and M2 modes can be converted with each other. The physical mechanism behind this reversible conversion is explained as follows. As discussed in Section 3.3.1, it is known that a path length of 5*a* causes a phase delay of about 2*k*π (*k* is integral) along the PEC surface. Here, the path-length-difference δ*L* between the upper and lower equivalent paths is 1.25a×2=2.5a (i.e., δ*L* = 2.5*a*), which causes a phase delay of (2*k −* 1)π (*k* is integral) and the mode conversion between M1 and M2 modes accordingly. Without loss of generality, here we set *k* = 1 (i.e., a path length of 2.5a will cause a phase delay of π), which will help the descriptions of the following discussions.

Similarly, for the cases of the waveguide B with a two-stranded coupling structure, they exhibit a similar phase delay effect as well. The M3 odd mode and M4 even mode can also accomplish a good reversible conversion between each other, as depicted inFigure 6c,d, where the M3 odd mode is transformed into the M4 even mode and vice versa. Moreover, as discussed above, the M4 mode is a slow-light TPS with a low GVD, whereas the M3 mode is a fast-light TPS with a high group velocity. Therefore, we can realize the conversion between fast- and slow-light states conveniently. It should be noted that we can realize the mode conversions between the M1/M2 or M3/M4 modes in two waveguides by inserting PECs with the same lengths that cause identical path-length-differences δ*L*, implying that the phase delay generated by PEC is independent of the parity of modes in waveguides, and it is dominated by the PEC itself and the surroundings bypassing PEC.

#### 3.4.2. Periodic Conversion of Modes

**Figure 7:**
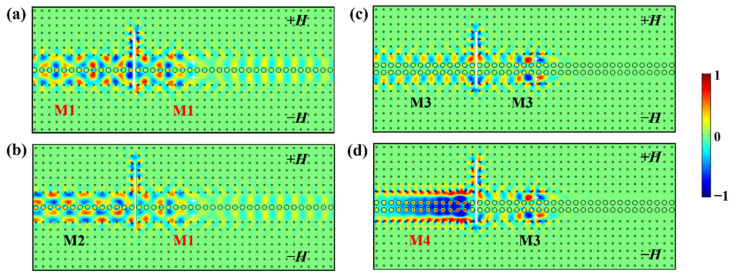
The *E_z_* field distributions with different lengths of PEC defect asymmetrically inserted in the waveguide. (**a**,**c**) 7.5*a*; (**b**,**d**) 8.75*a*.

Here, we further study the periodic mode conversion phenomenon. We take the M1 and M3 modes as examples and consider two cases where the increments of the upper part of the PEC are 2.5*a* and 3.75*a* (i.e., the length of the PEC defect is increased to 7.5*a* and 8.75*a*), leading to path-length-differences of 2δL and 3δL between the upper and lower equivalent paths, respectively. The simulation results are shown in Figure 7a–d, respectively.

From the case in Figure 7a,c, since the EM wave along the upper equivalent path travels a longer path length of 2δL than that along with the lower one, it will cause a phase delay of 2π so that no mode conversion occurs when the length of PEC is 7.5*a* for both the M1 and M3 modes. However, for the case in Figure 7b,d, the path-length-difference of 3δL results in a phase delay of 3π, which leads to odd/even mode conversion when the length of PEC is 8.75*a* accordingly. These results indicate that when δL is changed by a step of 2.5*a*, i.e., the increment or decrement of the upper part of the PEC is 1.25*a* × *m* (*m* is integral), the even/odd modes of conversion will alternately appear between conversion and non-conversion in the double-channel waveguide. In other words, the delay phase generated by δL plays a crucial role in the realization of mode conversion regardless of the length and location of PEC, which is consistent with our theoretical predictions.

#### 3.4.3. Validation of Continuous Reversible Mode Conversion

**Figure 8:**
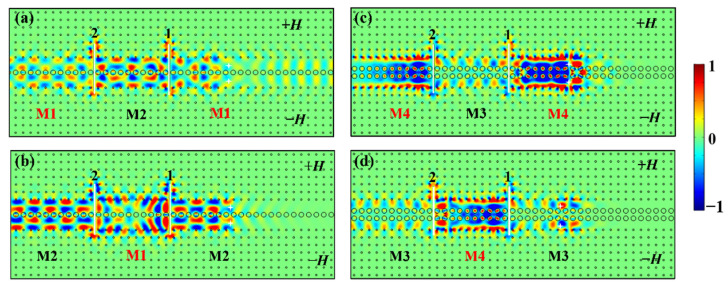
The *E_z_* field distributions with two PEC defects of 6.25*a* inserted into the one- and two-stranded waveguides for excited (**a**) M1 mode, (**b**) M2 mode, (**c**) M4 mode, (**d**) M3 mode.

To further discuss the phenomena of continuous reversible mode conversion, we insert two identical PECs of 6.25*a* into the one- and two-stranded waveguides, respectively. We excite the four modes separately to justify whether it can recover its initial mode after bypassing two PECs that trigger conversions twice between even and odd modes. The simulation results are shown in Figure 8.

For the case of the M1 mode in Figure 8a, the EM wave propagates leftwards in the waveguide. After climbing over the first PEC, the M1 even mode is converted into the M2 odd mode and continues to propagate in the form of odd mode before meeting the second PEC. Then it recovers into the M1 even mode after bypassing the second PEC, which provides another phase delay of π to realize the second mode conversion. There are mode conversions twice (M1−> M2−> M1) during the whole course. Similarly, the *E_z_* distributions for the M2, M3, and M4 modes demonstrate that they also undergo twice mode conversions (i.e., M2−> M1−> M2, M3−> M4−> M3, M4−> M3−> M4) and return to their original modes after bypassing the two PECs (Figure 8b–d). It is worth mentioning that during the reversible mode conversion between the M3 odd mode and M4 even mode, we have also realized the reversible conversion between slow-light and fast-light states in different regions of the waveguide by inserting PECs with suitable lengths, which have the potential to control light states.

### 3.5. Transmission Spectra Analysis of Double-Channel Waveguides

**Figure 9:**
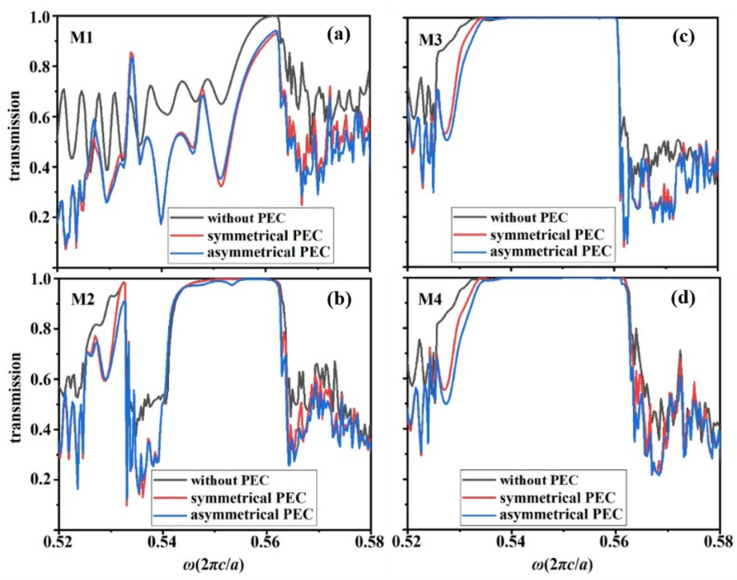
The transmission spectra for (**a**) M1 mode, (**b**) M2 mode, (**c**) M3 mode, and (**d**) M4 mode in three cases of without PEC, symmetric PEC and asymmetric PEC defect.

Furthermore, we explore the influence of PEC on energy transmittance in the double-channel structure. Figure 9 shows the calculated transmission spectra of the four modes in three cases without PEC, symmetrical PEC of 5*a*, and asymmetrical PEC of 6.25*a* defects. In Figure 9a, for the transmission spectrum of the M1 mode, the transmittance of the case without PEC defect is less than 1 due to the aforementioned physical reason that it inevitably causes the partial excitation of the red band curve near the right Brillouin boundary. Thus, no matter whether the PEC defect is inserted in the waveguide symmetrically or asymmetrically, the transmittance in the bandgap decreases significantly. Differently, in Figure 9b for the M2 mode, the transmission spectra for the cases without PEC and symmetric PEC are perfectly coincident to be near 100% within the bandgap, while that of the case of asymmetric PEC changes very slightly. As for the M3 and M4 modes in waveguide B, the transmission spectra of the three cases (i.e., without PEC, symmetrical PEC, and asymmetrical PEC defects) are totally coincident with each other. Their transmittances are 100% in the bandgap, revealing that the influence of PEC defects on energy transmittance is negligible and the energy robustness of the one-way waveguide is very strong.

## 4. Conclusions

In conclusion, we have investigated the topological transmission properties of odd/even one-way modes and their reversible conversion in double-channel MOPC waveguides. For waveguide A with one-stranded Al_2_O_3_ rods as the coupling layer, there appears an M1 even mode and an M2 odd mode, both of which are fast-light modes and result from the weak coupling effect of two sub-waveguide modes. Differently, for the waveguide B with two-stranded Al_2_O_3_ rods, there exists a fast-light mode (M3 odd mode) and a slow-light mode (M4 even mode), which are caused by the weak and strong coupling effects of two sub-waveguide modes, respectively. Furthermore, we have also realized the reversible conversion between the M1 and M2 modes, or the M3 and M4 modes, respectively, by adjusting the length of the PEC defect to achieve the desired phase delay difference along two equivalent transmission paths. What’s more, we find that although the robustness of the M1 mode is poor due to extra excitations of counter-propagation modes near the right Brillouin boundary, the M2, M3, and M4 modes have extremely strong robustness against PEC defects and their one-way transmittances are nearly 100%. These results have the potential for slow-light modulation and various topological device designs.

## Data Availability

The data presented in this study are available on request from the corresponding author.

## References

[1] Ozawa T., Price H.M., Amo A., Goldman N., Hafezi M., Lu L., Carusotto I. (2019). Topological photonics. Rev. Mod. Phys..

[2] Tang G., He X., Shi F., Liu J., Chen X., Dong J. (2022). Topological photonic crystals: Physics, designs and applications. Photonics Rev..

[3] Wang X., Zhao W., Zhang H., Elshahat S., Lu C. (2022). Magnetic-Optic Effect-Based Topological State: Realization and Application. Front. Mater..

[4] Liu H., Su Z., Zhang Z.-Q., Jiang H. (2020). Topological Anderson insulator in two-dimensional non-Hermitian systems. Chin. Phys. B.

[5] Qin M., Xiao S., Liu W., Ouyang M., Yu T., Wang T., Liao Q. (2021). Strong coupling between excitons and magnetic dipole quasi-bound states in the continuum in WS2-TiO2 hybrid metasurfaces. Opt. Express.

[6] Chen J., Liang W., Li Z.-Y. (2020). Revealing photonic Lorentz force as the microscopic origin of topological photonic states. J. Nanophotonics.

[7] He L., Gao Y.-F., Jiang Z., Wang L.-S., Zhou J., Xu X.-F. (2018). A unidirectional air waveguide basing on coupling of two self-guiding edge modes. Opt. Laser Technol..

[8] Yu Z., Veronis G., Wang Z., Fan S. (2008). One-Way Electromagnetic Waveguide Formed at the Interface between a Plasmonic Metal under a Static Magnetic Field and a Photonic Crystal. Phys. Rev. Lett..

[9] Raghu S., Haldane F.D.M. (2008). Analogs of quantum-Hall-effect edge states in photonic crystals. Phys. Rev. A.

[10] Haldane F.D.M., Raghu S. (2008). Possible realization of directional optical waveguides in photonic crystals with broken time-reversal symmetry. Phys. Rev. Lett..

[11] Wang Z., Chong Y., Joannopoulos J.D., Soljačić M. (2009). Observation of unidirectional backscattering-immune topological electromagnetic states. Nature.

[12] Chen J., Liang W., Li Z.-Y. (2019). Switchable slow light rainbow trapping and releasing in strongly coupling topological photonic systems. Photonics Res..

[13] Guglielmon J., Rechtsman M.C. (2019). Broadband Topological Slow Light through Higher Momentum-Space Winding. Phys. Rev. Lett..

[14] Schulz S.A., Upham J., O’Faolain L., Boyd R.W. (2017). Photonic crystal slow light waveguides in a kagome lattice. Opt. Lett..

[15] Li F.-F., Wang H.-X., Xiong Z., Lou Q., Chen P., Wu R.-X., Poo Y., Jiang J.-H., John S. (2018). Topological light-trapping on a dislocation. Nat. Commun..

[16] Chen J., Liang W., Li Z.-Y. (2020). Antichiral one-way edge states in a gyromagnetic photonic crystal. Phys. Rev. B.

[17] Zhou P., Liu G.-G., Yang Y., Hu Y.-H., Ma S., Xue H., Wang Q., Deng L., Zhang B. (2020). Observation of Photonic Antichiral Edge States. Phys. Rev. Lett..

[18] Fu J.-X., Lian J., Liu R.-J., Gan L., Li Z.-Y. (2011). Unidirectional channel-drop filter by one-way gyromagnetic photonic crystal waveguides. Appl. Phys. Lett..

[19] Liu K., Shen L., He S. (2012). One-way edge mode in a gyromagnetic photonic crystal slab. Opt. Lett..

[20] Poo Y., Wu R.-X., Lin Z., Yang Y., Chan C.T. (2011). Experimental Realization of Self-Guiding Unidirectional Electromagnetic Edge States. Phys. Rev. Lett..

[21] He C., Chen X.-L., Lu M.-H., Li X.-F., Wan W.-W., Qian X.-S., Yin R.-C., Chen Y.-F. (2010). Tunable one-way cross-waveguide splitter based on gyromagnetic photonic crystal. Appl. Phys. Lett..

[22] Zhang J., Zhao B., Xue Y., Zhou T., Yang Z. (2018). Coupling effect of topological states and Chern insulators in two-dimensional triangular lattices. Phys. Rev. B.

[23] Gao F., Gao Z., Shi X., Yang Z., Lin X., Xu H., Joannopoulos J.D., Soljačić M., Chen H., Lu L. (2016). Probing topological protection using a designer surface plasmon structure. Nat. Commun..

[24] Chen Z.-G., Mei J., Sun X.-C., Zhang X., Zhao J., Wu Y. (2017). Multiple topological phase transitions in a gyromagnetic photonic crystal. Phys. Rev. A.

[25] Yang B., Zhang H., Shi Q., Wu T., Ma Y., Lv Z., Xiao X., Dong R., Yan X., Zhang X. (2020). Details of the topological state transition induced by gradually increased disorder in photonic Chern insulators. Opt. Express.

[26] Chen J., Li Z.-Y. (2022). Prediction and Observation of Robust One-Way Bulk States in a Gyromagnetic Photonic Crystal. Phys. Rev. Lett..

[27] Zhuang S., Chen J., Liang W., Li Z.Y. (2021). Zero GVD slow-light originating from a strong coupling of one-way modes in double-channel magneto-optical photonic crystal waveguides. Opt. Express.

